# Maternal serum level of TNF-α in Nigerian women with gestational diabetes mellitus

**DOI:** 10.11604/pamj.2018.31.250.16989

**Published:** 2018-12-28

**Authors:** Abdullahi Mohammed, Ibrahim Sambo Aliyu

**Affiliations:** 1 Department of Chemical Pathology Gombe State University, Gombe, Nigeria; 2 Department of Chemical Pathology Ahmadu Bello University, Zaria, Nigeria

**Keywords:** Diabetes, gestational, Pregnancy, Tumor Necrosis factor-alpha, Body mass index

## Abstract

**Introduction:**

alterations in the circulating level of tumour necrosis factor-α (TNF-α) has been proposed to be involved in the pathogenesis of gestational diabetes mellitus (GDM), but its role is not completely understood, findings from studies done across different ethnic groups are often inconsistent. We carried out this study to determine maternal serum level of TNF-a and it's association with body weight status in a group of Nigerian women with GDM.

**Methods:**

a cross sectional analytical study conducted among 169 pregnant women, 85 with GDM and 84 with normal gestation. Diagnosis of GDM was made between 24-28 weeks gestation according to the WHO diagnostic criteria. Maternal serum level of TNF-α was measured and compared between the study groups.

**Results:**

maternal serum TNF-α level was significantly higher in the pregnant women with GDM than in the controls (2.50 ± 0.30 vs. 2.10 ± 0.30 pg/ml, p < 0.05). Also when comparing the serum TNF-α levels of the pregnant women with GDM and the controls for each level of body mass index, serum TNF-α levels remained significantly higher in both the normal weight and overweight pregnant women with GDM compared to their matched controls (2.40 ± 0.30 vs. 1.90 ± 0.20 pg/ml, p < 0.05) and (2.60 ± 0.30 vs. 2.30 ± 0.20 pg/ml, p < 0.05) respectively.

**Conclusion:**

it is concluded that pregnant women with GDM in this study have higher maternal serum TNF-α level compared to the pregnant women with normal glucose tolerance regardless of body weight status.

## Introduction

Gestational diabetes mellitus (GDM) is defined as any degree of glucose intolerance with onset or first recognition during pregnancy [1]. The prevalence of gestational diabetes mellitus has been increasing dramatically worldwide over the last two decades [2]. In Nigeria the prevalence ranges from 3.4-13.9% across the different regions of the country [3-7]. Gestational diabetes mellitus is associated with increased obstetric and perinatal morbidity and mortality [2]. Despite remarkable progress made in the field of endocrinology, the exact mechanism implicated in the pathogenesis of gestational diabetes mellitus is still not completely understood. Recently the role of inflammatory cytokines especially TNF-α has been increasingly investigated but findings are inconsistent especially across different ethnic groups [8-17]. While ethnicity is one of the factors that influence production of cytokines during pregnancy [18], studies on the association between alterations in the maternal circulating level of TNF-α and development of gestational diabetes mellitus among black African women are limited. We are not aware of any study done in Nigeria among women with gestational diabetes mellitus. This study was therefore designed to compare maternal circulating TNF-α levels between Nigerian pregnant women with and without gestational diabetes mellitus.

## Methods

A cross sectional analytical study that involved 85 pregnant women with gestational diabetes mellitus and 84 controls (pregnant women with normal glucose tolerance) at 24-28 weeks gestation. The study was conducted at Ahmadu Bello University Teaching Hospital, Zaria, Nigeria. Diagnosis of gestational diabetes mellitus was based on the WHO diagnostic criteria (2-hour 75g Oral glucose tolerance tests: fasting serum glucose ≥ 7.0mmol/L or 2-hour post load serum glucose ≥ 7.8mmol/L). Study subjects were recruited from the antenatal clinics of Ahmadu Bello University Teaching Hospital, Zaria and Hajiya Gambo Sawaba General Hospital Zaria, Kaduna State, Nigeria. Pregnant women with hypertension, history of pre gestational diabetes mellitus, multi foetal pregnancy, or any pre-existing illness were excluded from the study. The purpose of the study was explained fully to the participants, and written informed consent was obtained from each subject, before recruitment in to the study. The study was reviewed and approved by the Health Research Ethics Committee of Ahmadu Bello University Teaching Hospital, Zaria and Kaduna State Ministry of Health, Kaduna. History including maternal age, parity and gestational age were all obtained from the subjects at the time of enrolment. Gestational age was based on the report of ultrasound scan. Body mass index (BMI) was calculated as the ratio of weight in kilogram to square of height in meters and expressed as kg/m2. The two-hour 75g oral glucose tolerance test was performed in the morning (8:00am-10:30am) following 10-12 hours overnight fast. A fasting blood sample was drawn for measurement of fasting serum glucose and TNF-α. Two-hour blood sample after an oral glucose load was taken for measurement of 2-hour serum glucose. Serum samples were prepared and stored in a freezer (-20oC) until time of analysis.

**Biochemical analysis:** serum glucose was measured using an enzymatic glucose oxidase method (Labkit France). Serum TNF-α was assayed using human TNF-α ELISA kit (Wkea Med Supplies Corp. China). Samples were analysed at the Chemical Pathology Laboratory of Ahmadu Bello University Teaching Hospital, Zaria.

**Statistical analysis:** statistical analysis was performed using the SPSS version 20.0 statistical package. Data are summarised using measures of central tendency and dispersion. We compared mean differences of serum TNF-α level between groups using t-test. All p-values were 2-sided and considered significant if less than 0.05.

## Results

**Figure 1:**
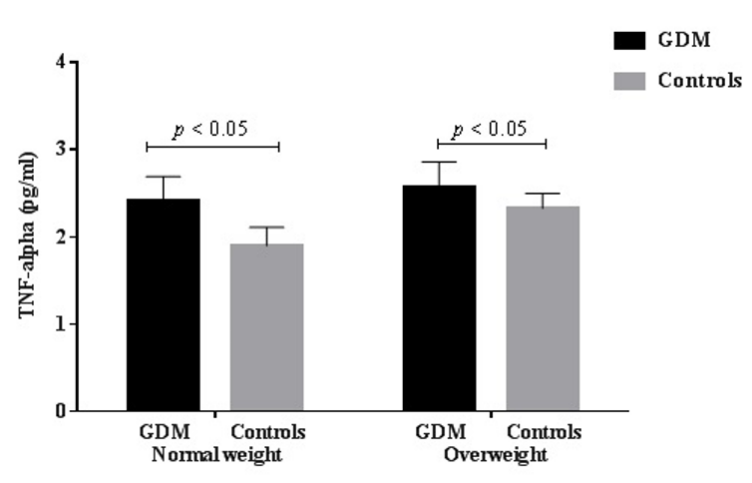
serum TNF-α levels among normal weight and overweight GDM subjects and controls

Demographic characteristics of the study subjects are presented in Table 1. Both the study groups were of similar maternal age, gestational age and parity (26.0 ± 6.0 vs. 27.0 ± 5.0 years, p = 0.041), (26.5 ± 1.0 vs. 26.0 ± 1.0 weeks, p = 0.196) and (2.3 ± 1.6 vs. 2.7 ± 1.7, p = 0.106) respectively. Pregnancy BMI of pregnant women with gestational diabetes mellitus was significantly higher than in the normal controls (25.4 ± 4.0 vs. 23.5 ± 3.8 kg/m2, p < 0.05). Biochemical parameters of the study subjects are shown in Table 2. Serum level of TNF-α was significantly higher among the pregnant women with gestational diabetes mellitus compared to the controls (2.50 ± 0.30 vs. 2.10 ± 0.30 pg/ml, p < 0.05). When the maternal serum TNF-α levels of the pregnant women with gestational diabetes mellitus and the controls were compared for each level of BMI (Table 3), serum TNF-α levels remained significantly higher in both the normal weight and overweight pregnant women with gestational diabetes mellitus compared to their matched controls (2.4 ± 0.3 vs. 1.9 ± 0.2 pg/ml, p < 0.05) and (2.6 ± 0.3 vs. 2.3 ± 0.2 pg/ml, p < 0.05) respectively (Figure 1).

**Table 1:** clinical characteristics of the study participants

Variables	GDM(m ± SD)	Controls(m ± SD)	*p*-value
Sample size (n)	85	84	
Age (years)	26.0 ± 6.0	27.0 ± 5.0	0.041
Parity	2.3 ± 1.6	2.7 ± 1.7	0.106
Gestational age (weeks)	26.0 ± 1.5	26.3 ± 1.4	0.196
Weight (kg)	65.0 ± 15.0	62.0 ± 14.0	0.124
Height (cm)	159.0 ± 8.0	161.0 ± 7.0	0.069
Body Mass Index BMI (kg/m^2^)	25.4 ± 4.0	23.5 ± 3.8	0.002
Systolic blood pressure (mmHg)	117.0 ± 8.0	118.0 ± 5.0	0.139
Diastolic blood pressure (mmHg)	78.0 ± 5.0	79.0 ± 3.0	0.296

m, Mean. SD, standard deviation. GDM, gestational diabetes mellitus

**Table 2:** Biochemical profiles of the study participants

Variables	GDM (m ± SD)	Controls (m ±SD)	*p*-value
Sample size(n)	85	84	
OGTT Fasting serum glucose (mmol/L)	4.3 ± 0.6	4.3 ± 0.7	0.439
2 hour serum glucose (mmol/L)	9.0 ± 0.9	6.3 ± 0.8	0.000
Serum Tumour necrosis factor-α (pg/ml)	2.5 ± 0.3	2.1 ± 0.3	0.000

m, Mean. SD, standard deviation. OGTT, oral glucose tolerance test. GDM, gestational diabetes mellitus

**Table 3:** biochemical profiles of the study participants in relation to body mass index

	GDM (m ± SD)	Controls (m ± SD)	*p*-value
**BMI <25kg/m^2^**	39	53	
Fasting serum glucose (mmol/L)	4.2 ± 0.7	4.1 ± 0.7	0.765
2 hour serum glucose (mmol/L)	9.0 ± 0.9	6.2 ± 0.8	0.000
TNF-α (pg/ml)	2.4 ± 0.3	1.9 ± 0.2	0.000
**BMI ³25kg/m^2^**	46	31	
Fasting serum glucose (mmol/L)	4.5 ± 0.6	4.5 ± 0.7	0.987
2 hour serum glucose (mmol/L)	9.1 ± 0.8	6.3 ± 0.8	0.000
TNF-α (pg/ml)	2.6 ± 0.3	2.3 ± 0.2	0.000

m, Mean. SD, standard deviation. BMI, body mass index. Ns, not significant. GDM, gestational diabetes mellitus

## Discussion

This study has shown that the pregnant women with gestational diabetes mellitus have higher serum TNF-α levels than the pregnant women with normal gestation. This finding of increased serum TNF-α level in pregnant women with gestational diabetes mellitus has been reported in many previous studies [8, 11- 13]. Also in line with the results of this study Laetitia *et al.* reported that higher maternal serum level of TNF-α is a significant predictor of developing gestational diabetes mellitus [3]. When comparing maternal serum TNF-α levels of the study subjects for each level of body mass index, serum TNF-α levels remained significantly higher in both the normal weight and overweight pregnant women with gestational diabetes mellitus compared to their matched controls. The same finding was reported by McLachlan *et al.* [9], although in a study with a relatively smaller sample size (19 GDM and 19 controls). However, the findings of Saucedo *et al.* did not show significant difference between serum TNF-α levels in pregnant women with gestational diabetes mellitus compared to normal controls. Their negative findings might be due to the differences in timing of maternal blood collection, assay methods used and ethnicity of the study subjects [10]. The discrepancy might also be due to the greater BMI of their study subjects (mean BMI of 30kg/m2 and 28kg/m2 for the GDM and control groups respectively). The finding of increased maternal inflammatory cytokine (TNF-α) among women with GDM as compared to those with normal gestation, in this study, further supports the previous reports that suggests GDM is associated with amplified inflammatory response.

## Conclusion

We conclude that among the pregnant women in this study, those with gestational diabetes mellitus have higher serum TNF-α levels than the pregnant women with normal glucose tolerance regardless of body weight status, suggesting that alterations in the circulating level of TNF-α might be related to the pathogenesis of gestational diabetes mellitus. We recommend larger prospective studies within the population that would examine the mechanism of alteration of the maternal circulating TNF-α level and its pattern from first through third trimester in pregnant women with gestational diabetes mellitus.

### What is known about this topic

- Gestational diabetes mellitus results from an imbalance between insulin resistance and insulin secretion capacity during pregnancy;
- Pro-inflammatory cytokines might cause insulin resistance by inhibiting insulin signal transduction.

### What this study adds

- The findings in this present study corroborates findings in previous reports suggesting that alterations in the circulating level of the inflammatory cytokine (TNF-a) might be related to the pathogenesis of gestational diabetes mellitus regardless of body weight status;
- The results of the study suggested that regulation of TNF-α may provide an important target for physiological and pharmacological interventions designed to reduce the risk of adverse pregnancy outcomes related to gestational diabetes. These may include prompt treatment of disease conditions that are associated with release of inflammatory cytokines including the TNF-α e.g. urinary tract infection.

## Competing interests

The authors declare no competing interests.
